# The structural connectivity of higher order association cortices reflects human functional brain networks

**DOI:** 10.1016/j.cortex.2016.08.011

**Published:** 2017-12

**Authors:** JeYoung Jung, Lauren L. Cloutman, Richard J. Binney, Matthew A. Lambon Ralph

**Affiliations:** a Neuroscience and Aphasia Research Unit (NARU), School of Biological Sciences, University of Manchester, UK; b Eleanor M. Saffran Center for Cognitive Neuroscience, Temple University, Philadelphia, PA, USA

**Keywords:** Associative cortex, Higher cognitive function, Diffusion weighted imaging, Tractography, Graph-theory, STG, superior temporal gyrus, LAT, lateral temporal pole, MED, medial temporal pole, MTG, middle temporal gyrus, ITG, inferior temporal gyrus, FG, fusiform gyrus, PhG, parahippocampal gyrus, HG, Heschl's gyrus, LG1, lingual gyrus next to fusiform gyrus, LG2, medial lingual gyrus, DLPFC, dorsolateral prefrontal cortex, OFC, orbitofrontal cortex, p.Op, pars opercularis, p.Tri, pars triangularis, p.Orb, pars orbitalis, BA, Brodmann's areas, IPS, intraparietal sulcus, 5Ci, 5M, 5L, BA 5 (superior parietal cortex), 7PC, 7A, 7P, 7M, BA 7 (superior parietal cortex), PFop, PFt, PF, PFcm, PFm, supramarginal gyrus, PGa, PGp, angular gyrus

## Abstract

Human higher cognition arises from the main tertiary association cortices including the frontal, temporal and parietal lobes. Many studies have suggested that cortical functions must be shaped or emerge from the pattern of underlying physical (white matter) connectivity. Despite the importance of this hypothesis, there has not been a large-scale analysis of the white-matter connectivity within and between these associative cortices. Thus, we explored the pattern of intra- and inter-lobe white matter connectivity between multiple areas defined in each lobe. We defined 43 regions of interest on the lateral associative cortex cytoarchitectonically (6 regions of interest – ROIs in the frontal lobe and 17 ROIs in the parietal lobe) and anatomically (20 ROIs in the temporal lobe) on individuals' native space. The results demonstrated that intra-region connectivity for all 3 lobes was dense and graded generally. In contrary, the inter-lobe connectivity was relatively discrete and regionally specific such that only small sub-regions exhibited long-range connections to another lobe. The long-range connectivity was mediated by 6 major associative white matter tracts, consistent with the notion that these higher cognitive functions arises from brain-wide distributed connectivity. Using graph-theory network analysis we revealed five physically-connected sub-networks, which correspond directly to five known functional networks. This study provides strong and direct evidence that core functional brain networks mirror the brain's structural connectivity.

## 1 Introduction

The frontal, temporal and parietal lobes contain the majority of the tertiary association cortex, which are key substrates for higher cognition including executive function, language, memory and attention. Each cognitive domain arises from coordinated action between a widespread, distributed neural network within these regions. For example, the executive control network is embedded in subsets of frontoparietal areas (Seeley et al., 2007), the episodic memory system relies on a network connecting medial temporal areas to parietal and frontal regions (Alvarez & Squire, 1994), and language functions arise from an extensive network including Broca's and Wernicke's areas, as well as other prefrontal, temporal and parietal regions (Binder et al., 1997). Although other subcortical structures such as basal ganglia (Leisman, Braun-Benjamin, & Melillo, 2014) and thalamus (Mitchell et al., 2014) also contribute to these cognitive functions, we focused on cortico-cortical pathways between the major associative cortices in the current study.

Evidence spanning from lesion studies to functional connectivity have mapped functional networks by linking each cognitive activity to individual regions within a brain network. Multiple researchers have noted that the contributions of each brain region to large-scale network functions must be heavily shaped by their structural connectivity (Friston, 2002, Mesulam, 1990, Passingham et al., 2002, Sporns et al., 2005). Thus, it becomes necessary to investigate the white matter pathways that connect cortical areas in order to understand how each cognitive activity arises from the patterns of brain-wide distributed connectivity.

Diffusion neuroimaging and tractography methods allow researchers to reveal white matter fibre structure and to map white matter cortico-cortical projections at high spatial resolution, *in vivo* and *en masse* (Conturo et al., 1999, Parker and Alexander, 2005). Such studies generate a matrix of inter-regional connectivity which can be further explored using mathematical techniques such as graph-theory (for the review, see Bullmore and Sporns, 2009, Gong et al., 2009, Hagmann et al., 2007, Iturria-Medina et al., 2008). Previous diffusion neuroimaging studies have tended to focus on either reconstructed major associative fasciculi (Catani and Thiebaut de Schotten, 2008, Makris et al., 2009) or have demonstrated topological properties within discrete targeted structural networks, with particular reference to primary sensory and motor regions/function (Gong et al., 2009, Hagmann et al., 2007). In addition, most studies using these methods have not yet fully covered the whole brain owing to susceptibility-induced geometric distortion of the MRI signal which leads to erroneous fibre tracking (Embleton, Haroon, Morris, Lambon Ralph, & Parker, 2010). This is particularly problematic around the rostral temporal cortices which are known to be important for semantic memory, language and visual processes (Binney et al., 2012, Shimotake et al., 2015). Therefore, the current study utilised targeted diffusion datasets that overcome the magnetic susceptibility artefacts by adopting new and advance DWI and tractography methodologies (Embleton et al., 2010, Haroon et al., 2009, Jeurissen et al., 2011, Parker and Alexander, 2005) (see the Materials and methods for the details).

In the current study, we explored the pattern of intra- and inter-lobe white matter connectivity between multiple areas defined within each lobe. In order to examine this large-scale frontal, temporal and parietal network, regions of interest (ROIs) were defined anatomically in temporal lobe (20 ROIs covering from anterior to posterior temporal cortices) and cytoarchitectonically in lateral frontal (6 ROIs) and parietal lobe (17 ROIs). To map the connectivity among ROIs systematically, we employed probabilistic tractography of distortion-corrected diffusion-weighted imaging at high angular resolution, which overcomes the signal dropout and image distortion within anteroventral temporal areas (Embleton et al., 2010, Parker and Alexander, 2005). In addition, graph-theory network analysis was conducted to quantify the network properties in our tractography results and thus reveal the underlying topology of the intra/inter-regional structural connectivity for frontal, temporal and parietal lobes. To the best of our knowledge, this is the first attempt to look into the structural patterns of connectivity of specifically targeted sub-regions that cover the majority of the human tertiary association cortices.

## 2 Materials and methods

### 2.1 Participants

Twenty-four participants (11 females; mean age = 25.9, range = 19–47) participated in this study, which was approved by the local ethics boards. All were right-handed as assessed by the Edinburgh Handedness Inventory (Oldfield, 1971). Written informed consent was obtained from all participants.

### 2.2 Diffusion weighted imaging and distortion correction

Imaging data were acquired on a 3-T Philips Achieva scanner (Philips Medical System, Best, Netherlands), using an 8 element SENSE head coil. Diffusion weighted imaging was performed using a pulsed gradient spin echo-planar sequence, with TE = 59 msec, TR ≈ 11,884 msec, *G* = 62 mTm^−1^, half scan factor = .679, 112 × 112 image matrix reconstructed to 128 × 128 using zero padding, reconstructed resolution 1.875 × 1.875 mm, slice thickness 2.1 mm, 60 contiguous slices, 61 non-collinear diffusion sensitization directions at *b* = 1200 s mm^−2^ (Δ = 29.8 msec, *δ* = 13.1 msec), 1 at *b* = 0, SENSE acceleration factor = 2.5. Acquisitions were cardiac gated using a peripheral pulse unit positioned over the participants' index finger or an electrocardiograph. For each gradient direction, two separate volumes were obtained with opposite polarity *k*-space traversal with phase encoding in the left-right/right-left direction to be used in the signal distortion correction procedure (Embleton et al., 2010). A co-localized T2 weighted turbo spin echo scan, with in-plane resolution of .94 × .94 mm and slice thickness 2.1 mm, was obtained as a structural reference scan to provide a qualitative indication of distortion correction accuracy. A high resolution T1-weighted 3D turbo field echo inversion recovery image (TR ≈ 2000 msec, TE = 3.9 msec, TI = 1150 msec, flip angle 8°, 256 × 205 matrix reconstructed to 256 × 256, reconstructed resolution .938 × .938 mm, slice thickness .9 mm, 160 slices, SENSE factor = 2.5), was also acquired for the purpose of high-precision construction of anatomically based ROIs.

Some existing diffusion datasets suffer from susceptibility-induced geometric distortion of the MRI signal which leads to erroneous fibre tracking (Embleton et al., 2010). This is particularly problematic around the rostral temporal cortices which are known to be important for semantic memory, language and visual processes (Binney et al., 2012, Shimotake et al., 2015). In current study, we reduced the magnetic susceptibility artefacts by adopting specific DWI and tractography methodologies (Embleton et al., 2010, Haroon et al., 2009, Jeurissen et al., 2011, Parker and Alexander, 2005).

### 2.3 Definition of regions of interest

**Fig. 1:**
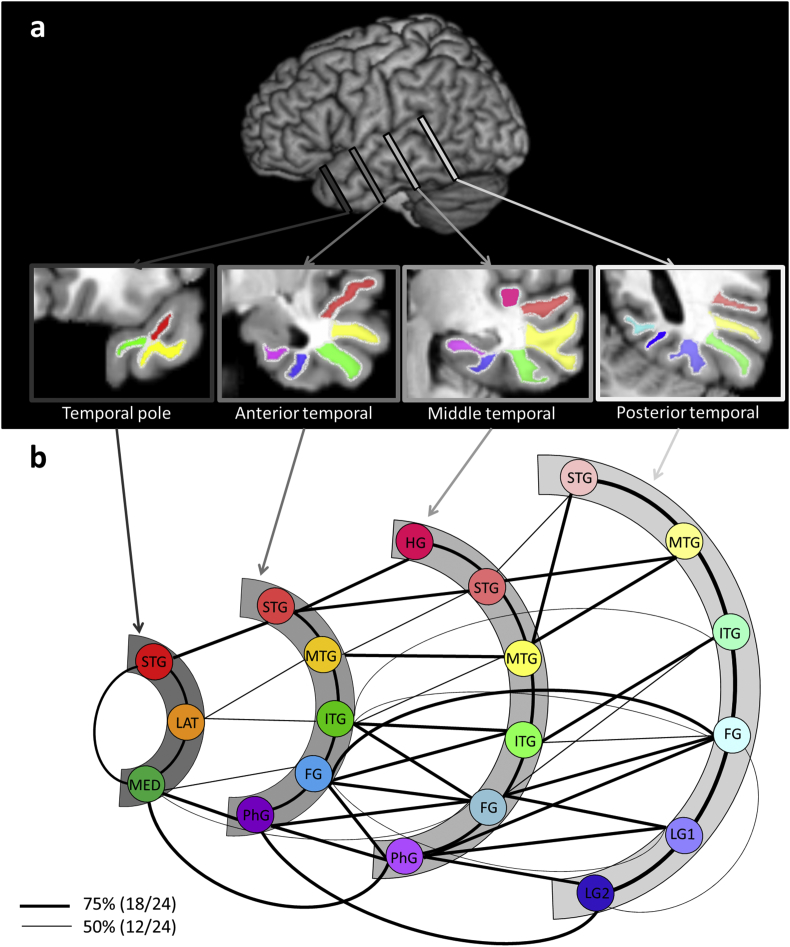
Intratemporal lobe connectivity. Each temporal lobe cross-section is represented by an arc that had been colour coded to indicate its position (the darker the grey, the more anterior). Each ROI within a cross-section is represented by a circle. Lines connecting ROIs are displayed if the probabilistic tractography exceeded the minimum probability threshold [2.5% of the total combined number of streamlines propagated from the two regions (the pathways detected at a more stringent threshold of 5% are displayed in Supplementary Figure S1)] in either 50% or 75% of the participants. (A) The example of the 21 temporal lobe regions of interest used for probabilistic tractography. (B) Summary of intratemporal lobe connectivity. Each coronal slice is represented by an arc. The temporal lobe regions within each slice are represented by circles. Connecting lines illustrate white matter pathways between pairs of regions survived at the thresholded connectivity matrix. Thick lines represent white matter pathways thresholded at 75% and thin lines thresholded at 50% from group analysis.

**Fig. 2:**
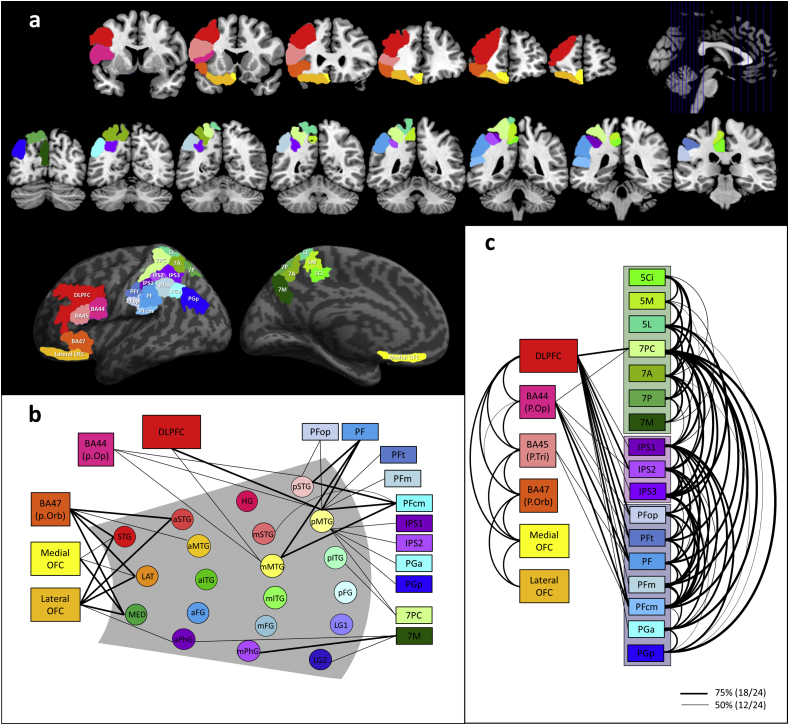
(A) The frontal and parietal regions of interest. (B) Extratemporal lobe connectivity. Temporal lobe regions are represented as circles within the grey part which has the same arrangement for the temporal area as Fig. 1b but without showing the intra-temporal connections. The frontal and parietal regions are represented as boxes, each with a unique colour. a = anterior temporal; m = middle temporal; p = posterior temporal. (C) Fronto-parietal lobe connectivity. Connecting lines illustrate white matter pathways between pairs of regions survived at the thresholded connectivity matrix. Thick lines represent white matter pathways thresholded at 75% and thin lines thresholded at 50% from group analysis.

Rather than tracking from large areas and thus potentially losing detailed information and important variations in connectivity profile, the large cortical regions (temporal lobe, parietal lobe and ventral/lateral prefrontal cortex) were split into a collection of small ROI. In the case of parietal and frontal regions, detailed cytoarchitectural maps are now available (Eickhoff et al., 2005) and thus we utilised these as ROIs (Fig. 2a). Unfortunately the same type of fine anatomical subdivisions are not available for the temporal lobe (only the large Brodmann areas with no rostral-caudal distinctions) and thus following a previous investigation (Binney et al., 2012) we divided the temporal lobe into 20 ROIs according to anatomical landmarks in native space. ROIs for temporal lobe were drawn on each individual's T1 weighted anatomical imaging using MRIcro. Twenty temporal lobe regions covered a polar, anterior, middle and posterior cross-section of the left temporal lobe. These cross-sections were identified in each individual's scan on the basis of structural landmarks. First, the pitch of the scan was rotated at the anterior commissure by 20° so that the images of the scan became axially coplanar with the longitudinal axis of the temporal lobe (e.g., the length of the STG – superior temporal gyrus). As a result, all cross-sections were perpendicular to this axis. The temporal polar cross-section was defined by selecting the coronal slice 10 mm back from the anterior tip of the left temporal lobe. The middle temporal cross-section was defined as the coronal slice at which the inferior aspect of the superior cerebellar peduncle meets the posterior wall of the pons. The position that fell halfway between these slices was defined as the anterior cross-section and was invariably at the position of the basal artery. This participant-specific measurement (half of the distance between the middle and polar section) was used to define the location of the posterior temporal cross-section by applying it posteriorly to the middle cross-section. Examples of these slice positions are illustrated in Fig. 1a.

The white matter of each temporal gyri in the four cross-sections was delineated as an individual ROI. In the temporal polar cross-section, there were three ROIs, the white matter of ventromedial gyrus, the lateral gyrus and the superior (or dorsal medial) gyrus of temporal pole. The anterior temporal and middle temporal cross sections included the white matter of the STG, the middle temporal gyrus (MTG), the inferior temporal gyrus (ITG), the fusiform gyrus (FG) and parahippocampal gyrus (PhG). The middle temporal cross-section also included the white matter of Heschl's gyrus. In the posterior temporal cross-section, the regions included the white matter of the STG, the MTG, the ITG, the FG and a lateral lingual gyrus and a more medial lingual gyrus. Each white matter region was delineated from that of the temporal stem by a line drawn between fundi of each adjacent sulcus. All gyral tissue within this boundary was marked. This process was repeated on four contiguous coronal slices, resulting in a three-dimensional ROI mask (4 voxels wide in the T1 images, 2 in the diffusion weighted images). To make sure that only white matter voxels were included, the ROI was treated with a scaled image intensity filter (minimum threshold, 158; maximum threshold, 254).

The frontal lobe ROIs (see Fig. 2a) included medial orbitofrontal cortex (medOFC), lateral orbitofrontal cortex (latOFC), BA 44 (pars opercularis), BA 45 (pars triangularis), BA 47 (pars orbitalis), BA 46 and BA 9 (together forming dorsolateral prefrontal cortex). Probabilistic cytoarchitectonic maps from the SPM Anatomy toolbox (Eickhoff et al., 2005) were used as masks for BA 44 and BA 45 to ensure that the ROIs included predominantly the cortices and a small amount of underlying gyral white matter. The orbitofrontal ROIs were defined using AAL atlas masks. The other frontal ROIs were defined using Brodmann grey matter masks provided by the Wake Forest University Pickatlas toolbox.

The parietal ROIs covered superior parietal lobule (SPL), intraparietal sulcus (IPS) and inferior parietal lobule (IPL). Probabilistic cytoarchitectonic maps from the SPM Anatomy toolbox (Eickhoff et al., 2005) were used as masks to ensure that the ROIs included predominantly the cortices and a small amount of underlying gyral white matter. Therefore, there were seven ROIs in SPL (5L, 5M, 5Ci, 7A, 7PC, 7M, 7P), three in IPS (IPS1, IPS2, IPS3), and seven in IPL (PFop, PFt, PF, PFm, PFcm, PGa, PGp). All ROIs were resized according to their own probability (ranging from 60% to 90%) in order to avoid overlap between them (see Fig. 2a).

The diffeomorphic anatomical registration through an exponentiated lie algebra (DARTEL) toolbox (Ashburner, 2007) was used to transform the extra-temporal ROIs from anatomical MNI space into each individual's native diffusion space. The transform was estimated using each subject's T1-weighted image having first been co-registered to their diffusion weighted images. The accuracy of the transformation of ROIs into native space was also inspected using these anatomical images.

### 2.4 Probabilistic fibre tracking

A whole-brain volume of probability density functions (PDFs) was generated by analysing each individual's distortion corrected DWI data using constrained spherical deconvolution (CSD) (Tournier et al., 2008) and model-based residual bootstrapping (Haroon et al., 2009, Jeurissen et al., 2011). The CSD algorithm resolves multiple intravoxel fibre orientations and the application of a bootstrapping technique provides quantification of the uncertainty of the inferred fibre orientation. Thus, PDFs describe the uncertainty in the orientation(s) of fibre populations within a voxel.

Unconstrained probabilistic tractography was performed using the PICo software package (Parker & Alexander, 2005). 20,000 Monte Carlo streamlines were initiated from each voxel in each frontal, temporal, and parietal ROI. Step size was set to .5 mm. Stopping criteria for the streamlines were set so that tracking terminated if pathway curvature over a voxel was greater than 180°, or the streamline reached a physical path limit of 500 mm. It is perhaps worth noting here that given the number and volume of ROIs, repeated in all participants, that this process requires a considerable amount of processing time (around four months in total).

A single whole-brain probabilistic map was generated for each of the 43 ROIs for each participant. Probability maps were masked with each ROI and the maximum connectivity value (ranging from 0 to 20,000) was extracted. Thereby, we obtained a single probability estimate of a pathway between each pair of regions. These values were placed into an individual-specific matrix. The matrix contained two probability estimates for each pair of regions because tracking was performed in both directions (e.g., region A to region B and region B to region A). We combined these two probability estimates to form a single probability estimate for each pair of regions and for each participant. Then, the connectivity matrices were subjected to a double threshold to ensure that only connections with high probability in the majority of participants were considered. For the first-level individual threshold, following the approach described by Cloutman, Binney, Drakesmith, Parker and Lambon Ralph (2012), the *λ*-value of the Poisson distribution identified was used to determine a threshold value at *p* = .05. Across participants this fell between 2.5% and 5% of the total number of streamlines. For the second-level group threshold, we used both a stringent (over 75% of participants, i.e., at least 18/24 participants) and a more relaxed (over 50% of participants, i.e., at least 12/24 participants) criteria for consistency.

### 2.5 Graph-theory network analysis

A graph-theory approach was used to explore structural connectivity across frontal, temporal and parietal areas (Rubinov & Sporns, 2010). We assessed network properties to quantify the underlying topological structure of brain network. An adjacency matrix of ROIs (nodes) and connections (edges) represented the probabilistic connectivity values from the group-level analysis with 2.5% streamline/50% participant double threshold. Thus, the matrix comprised 43 nodes and 43 × 43 edge binary values.

Modularity is based on the difference between the number of edges found within modules and the number of edges predicted to lie within modules if all edges in the network were distributed at random. Therefore, this modularity measure quantifies the strength of division of a network into modules and is optimized to detect modules in the network.

To quantify which nodes play important roles within a network, we computed three different measures of node centralities. Degree centrality or the degree of a node was calculated by summing all edges connected to a node. The second measure was the betweenness centrality of a node. Betweenness centrality is defined as the fraction of all shortest paths between any pair of nodes that travel through that node. It is a useful measure to quantify how much information passes through a certain part of a network based on an assumption that optimal paths are used. Therefore, high betweenness centrality implies that nodes are crucial hubs and/or bridges in a network. Finally, the closeness centrality of a node was calculated as the inverse average path length of a node to all other nodes in a network. High closeness centrality represents that the node can reach any other node in a network efficiently and hence plays an important role in integrating the information within the network. The network analysis was computed by the Brain Connectivity Toolbox (Rubinov & Sporns, 2010).

## 3 Results

### 3.1 The patterns of intra- and inter-lobe white matter connectivity

The first goal of this study was to map the inter- and intra-lobe white matter connectivity of temporal, frontal and parietal regions using unconstrained probabilistic tractography. Fig. 1a shows the 20 ROIs covering the whole temporal lobe anteriorly and posteriorly. The intra-temporal lobe connectivity matrix is displayed in Table 1, with each entry representing the probability of a pathway (group level analysis), and is visualized in Fig. 1b. Each temporal lobe cross-section is represented by an arc that had been colour coded to indicate its position (the darker the grey, the more anterior). Each ROI within a cross-section is represented by a circle. Lines connecting ROIs are displayed if the probabilistic tractography exceeded the minimum probability threshold [2.5% of the total combined number of streamlines propagated from the two regions (the pathways detected at a more stringent threshold of 5% are displayed in Supplementary Fig. S1)] in either 50% or 75% of the participants.

**Table 1:** Group-level intratemporal lobe connectivity matrix. Bold font indicates that the connection probability was over 50% (12/24) for group analysis. The individual threshold was set at 2.5%.

		Temporal pole			Anterior temporal					Middle temporal						Posterior temporal					
		STG	LAT	MED	STG	MTG	ITG	FG	PhG	HG	STG	MTG	ITG	FG	PhG	STG	MTG	ITG	FG	LG1	LG2
Temporal Pole	STG		**91.7**	**62.5**	**83.3**	45.8	8.3	4.2	8.3	8.3	4.2	29.2	8.3	8.3	20.8	4.2	8.3	16.7	12.5	12.5	12.5
	LAT			**91.7**	20.8	**66.7**	**70.8**	16.7	25.0	0	0	41.7	37.5	25.0	41.7	12.5	12.5	29.2	45.8	45.8	12.5
	MED				0	12.5	37.5	**58.3**	**91.7**	0	0	8.3	20.8	50.0	**83.3**	0	0	25.0	33.3	25.0	33.3

Anterior temporal	STG					**91.7**	8.3	0	0	**87.5**	**87.5**	33.3	8.3	0	0	16.7	4.2	16.7	29.2	0	0
	MTG						**95.8**	4.2	0	4.2	**58.3**	**91.7**	41.7	0	0	20.8	20.8	16.7	12.5	8.3	0
	ITG							**91.7**	8.3	8.3	4.2	**70.8**	**91.7**	**79.2**	45.8	16.7	29.2	**62.5**	54.2	29.2	0
	FG								**95.8**	0	0	12.5	**83.3**	**100**	**91.7**	0	8.3	37.5	**79.2**	**54.2**	45.8
	PhG									0	0	0	12.5	**75.0**	**100**	0	4.2	8.3	29.2	45.8	**91.7**

Middle temporal	HG										**100**	**58.3**	12.5	0	0	20.8	0	4.2	8.3	0	0
	STG											**100**	0	0	4.2	**66.7**	12.5	4.2	8.3	0	0
	MTG												**91.7**	0	0	**91.7**	**79.2**	33.3	0	8.3	0
	ITG													**95.8**	25.0	16.7	41.7	**75.0**	**62.5**	12.5	0
	FG														**95.8**	0	12.5	**70.8**	**100**	**79.2**	**66.7**
	PhG															0	0	8.3	**75.0**	**95.8**	**100**

Posterior temporal	STG																**95.8**	16.7	0	0	0
	MTG																	**100**	29.2	0	0
	ITG																		**100**	8.3	0
	FG																			**100**	**66.7**
	LG1																				**100**
	LG2																				

STG = superior temporal gyrus; LAT = lateral temporal pole; MED = medial temporal pole; MTG = middle temporal gyrus; ITG = inferior temporal gyrus; FG = fusiform gyrus; PhG = parahippocampal gyrus; HG = Heschl's gyrus; LG1 = lingual gyrus next to fusiform gyrus; LG2 = medial lingual gyrus.

The result of the intra-temporal connectivity replicated and extended the previous findings from Binney et al. (2012); there is strong connectivity down the length of each temporal gyrus and there is considerable lateral connectivity from each temporal gyrus to its neighbours cross-sectionally as well as diagonally. All connections found in the temporal lobe reflect a combination of short U-shaped fibres between gyri plus the middle longitudinal fasciculus (MdLF) and the inferior longitudinal fasciculus (ILF). The MdLF passes through STG from the temporal pole to posterior STG, Hechl's gyrus and IPL regions (Makris et al., 2009, Schmahmann et al., 2007). The ILF runs the length of the ventral temporal lobe connecting to occipital areas (Catani and Thiebaut de Schotten, 2008, Schmahmann et al., 2007) and its intra-temporal U-shaped fibres between gyri have been described as the occipito-temporal projection (Catani et al., 2003, Tusa and Ungerleider, 1985). Overall, these patterns of intra-lobe connectivity indicate that information primarily converges (1) laterally, towards MTG from superior and ventromedial regions and (2) longitudinally, toward temporal polar regions from posterior parts of temporal lobe (Binney et al., 2012). A final observation to note is that we did not find evidence for ‘diagonal’ connections between MTG and ITG in the middle temporal and the posterior temporal cross-sections. This might reflect the divergence of the major white matter pathways as they course into the parietal versus occipital lobe.

**Fig. 3:**
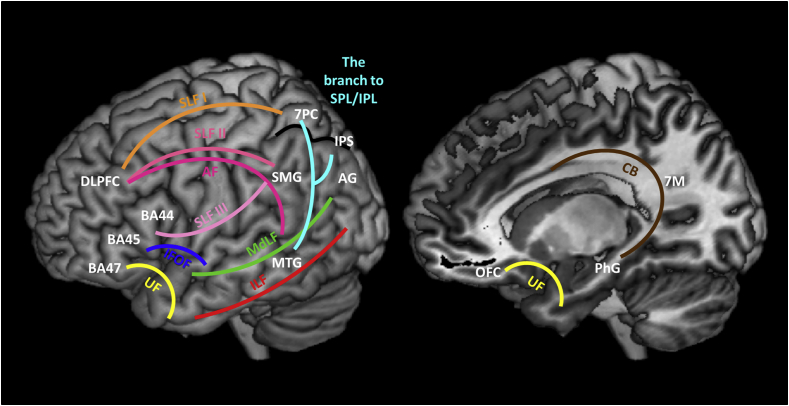
White matter tracks connecting frontal, temporal, and parietal lobe. The cyan coloured tack is a part of the other major tracks (IFOF, LIF or MdLF) connecting the posterior temporal region to the parietal cortices. The pink coloured tracks (AF/SLF II/III) are displayed separately as a matter of convenience but it is noted that they are not separable in the current study (see the detailed description of the main text). SMG: supramarginal gyrus including PFt, PF, PFop, and PFcm; AG: angular gyrus including PGa and PGp.

Fig. 2a shows the frontal and parietal ROIs, each with a unique colour. We mapped the white matter connectivity between the 20 temporal ROIs and these frontal and parietal sub-regions. The extra-temporal lobe connectivity matrix is summarised in Table 2 and illustrated in Fig. 2b. Unlike the graded intra-lobe connectivity, the extra-temporal connectivity exhibits regional-specific patterns of connectivity to the frontal and parietal areas. The temporal polar regions are connected to medial and lateral OFC as well as BA 47 (pars orbitalis) through the uncinate fasciculus (UF). In addition, anterior parahippocampal gyrus (aPhG) shared this same pathway to connect with lateral OFC. These tractography results are consistent with previous descriptions of the UF: a distinctive hook shape, long-range fasciculus connecting temporal pole with ventral frontal cortex (Gloor, 1997, Schmahmann et al., 2007). STG and MTG at the anterior temporal cross-section were exclusively linked with BA 47 via the inferior fronto-occipital fasciculus (IFOF), which connects temporal areas to ventral/lateral prefrontal cortices (Fig. 3) passing through the extreme capsule complex (Martino et al., 2010, Schmahmann et al., 2007).

**Table 2:** Group-level extratemporal lobe connectivity matrix. Bold font indicates that the connection probability was over 50% (12/24) for group analysis. The individual threshold was set at 2.5%.

		Frontal lobe						Superior parietal lobule							Inferior parietal sulcus			Inferior parietal lobule						
		DLPFC	BA 44	BA 45	BA 47	medOFC	latOFC	5Ci	5M	5L	7PC	7A	7P	7M	IPS1	IPS2	IPS3	PFop	PFt	PF	PFm	PFcm	PGa	PGp
Temporal pole	STG	8.3	4.2	8.3	**87.5**	**66.7**	**87.5**	0	0	0	8.3	4.2	0	0	4.2	0	4.2	0	4.2	0	0	0	0	0
	LAT	8.3	8.3	8.3	**87.5**	**58.3**	**83.3**	0	0	0	4.2	0	0	0	4.2	0	4.2	0	0	4.2	4.2	8.3	0	0
	MED	0	4.2	0	**87.5**	**87.5**	**95.8**	0	0	0	0	0	0	0	0	0	0	0	0	0	0	0	0	0

Anterior temporal	STG	4.2	25.0	33.3	**95.8**	8.3	**87.5**	0	0	16.7	29.2	8.3	8.3	20.8	8.3	0	8.3	4.2	0	12.5	0	20.8	4.2	12.5
	MTG	12.5	25.0	12.5	**50.0**	4.2	37.5	0	0	4.2	8.3	16.7	0	8.3	4.2	4.2	8.3	4.2	0	16.7	0	25.0	0	8.3
	ITG	20.8	25.0	16.7	12.5	0	12.5	0	0	0	8.3	4.2	4.2	4.2	8.3	8.3	4.2	4.2	0	25.0	8.3	25.0	4.2	12.5
	FG	0	0	0	0	0	0	0	0	0	0	0	0	25.0	0	0	0	0	0	4.2	0	0	0	0
	PhG	0	0	0	45.8	4.2	**66.7**	4.2	0	0	8.3	4.2	4.2	**66.7**	0	0	0	0	0	0	0	0	0	0

Middle temporal	HG	12.5	20.8	0	20.8	0	12.5	4.2	0	25.0	33.3	45.8	37.5	37.5	8.3	0	20.8	4.2	0	8.3	0	33.3	4.2	16.7
	STG	8.3	25.0	12.5	16.7	0	12.5	4.2	4.2	16.7	29.2	37.5	29.2	33.3	8.3	0	16.7	29.2	4.2	16.7	0	**62.5**	4.2	12.5
	MTG	**50.0**	**66.7**	33.3	45.8	0	29.2	0	4.2	12.5	37.5	29.2	20.8	12.5	25.0	12.5	20.8	37.5	12.5	**83.3**	20.8	**79.2**	33.3	16.7
	ITG	25.0	37.5	16.7	16.7	0	20.8	4.2	0	0	12.5	4.2	4.2	8.3	12.5	4.2	0	0	0	25.0	12.5	33.3	12.5	20.8
	FG	0	4.2	0	4.2	0	4.2	0	0	0	4.2	0	0	4.2	0	0	4.2	0	0	4.2	0	4.2	0	8.3
	PhG	0	0	0	20.8	0	45.8	0	0	0	0	16.7	8.3	**95.8**	0	0	0	0	0	0	0	0	0	0

Posterior temporal	STG	29.2	**50.0**	16.7	12.5	0	8.3	0	0	0	4.2	0	0	4.2	12.5	12.5	0	**62.5**	8.3	**91.7**	33.3	**79.2**	41.7	12.5
	MTG	**79.2**	**50.0**	37.5	4.2	0	8.3	0	0	4.2	**62.5**	4.2	0	0	**54.2**	**66.7**	20.8	**50.0**	**50.0**	**87.5**	**62.5**	**75.0**	**70.8**	**62.5**
	ITG	45.8	37.5	29.2	25.0	0	8.3	0	4.2	8.3	**58.3**	20.8	4.2	8.3	45.8	25.0	29.2	8.3	25.0	41.7	25.0	**54.2**	41.7	45.8
	FG	4.2	0	4.2	37.5	0	37.5	0	0	0	8.3	4.2	4.2	4.2	0	0	4.2	0	0	0	0	0	0	8.3
	LG1	0	0	0	16.7	8.3	20.8	0	0	0	0	0	0	33.3	0	0	0	0	0	0	0	0	0	0
	LG2	0	0	0	4.2	0	4.2	0	0	0	0	0	4.2	**62.5**	0	0	0	0	0	0	0	0	0	0

DLPFC = dorsolateral prefrontal cortex; BA = Brodmann's areas; medOFC = medial orbitofrontal cortex; latOFC = lateral orbitofrontal cortex; p.Op = pars opercularis; p.Tri = pars triangularis; p.Orb = pars orbitalis; IPS = intraparietal sulcus; 5Ci, 5M, 5L = BA 5 (superior parietal cortex); 7PC, 7A, 7P, 7M = BA 7 (superior parietal cortex); PFop, PFt, PF, PFcm, PFm = supramarginal gyrus; PGa, PGp = angular gyrus; STG = superior temporal gyrus; LAT = lateral temporal pole; MED = medial temporal pole; MTG = middle temporal gyrus; ITG = inferior temporal gyrus; FG = fusiform gyrus; PhG = parahippocampal gyrus; HG = Heschl's gyrus; LG1 = lingual gyrus next to fusiform gyrus; LG2 = medial lingual gyrus.

Dorsal-posterior temporal regions exhibited two distinctive pathways: connectivity to the frontal lobe and a pathway to the parietal lobe. The pathway to the frontal lobe has been reported in previous studies and is commonly attributed to arcuate fasciculus (AF) (Saur et al., 2008). Consistent with this view, our results also identified AF linking STG and MTG, at the middle/posterior temporal sections, with DLPFC and BA 44. In addition, we also observed evidence of the ‘ventral language pathway’ connecting pMTG to prefrontal cortex (Parker et al., 2005, Saur et al., 2008). The other pathways found in our tractography strongly connected posterior temporal regions to parietal cortices. First, the middle/posterior temporal STG and MTG connected to the supramarginal gyrus (PFt, PFop, PF, PFm, PFcm) via either AF or MdLF (Makris et al., 2013). Second, only the posterior MTG connected to the angular gyrus (PGa, PGp) and IPS, which appears to be attributable to the MdLF (Makris et al., 2009, Makris et al., 2013). Third, we observed a pathway linking posterior MTG and ITG with SPC (7PC), which may correspond to the parietal branch of the ILF (Schmahmann and Pandya, 2006). Fourth, basal-medial temporal areas showed direct connections to 7M, (precuneus in SPC) via the posterior part of the cingulum bundle (CB) (Mufson and Pandya, 1984, Schmahmann et al., 2007). Finally, we found no evidence of direct connections to frontal or parietal regions from ventral-lateral anterior temporal regions (Binney et al., 2012).

The fronto-parietal connectivity matrix is displayed in Table 3 and Fig. 2c. Within each lobe, all ROIs were highly connected with each other, whereas the inter-lobular connectivity was regionally specific. DLFPC and BA44 were directly connected to 7PC, which can be attributed to the superior longitudinal fasciculus (SLF I) (Catani et al., 2005, Makris et al., 2005). These frontal regions were also linked with IPS through the AF/SLF III (Makris et al., 2005). Our tractography revealed direct pathways between lateral frontal regions (DLPFC & Broca's areas) and the supramarginal gyrus (PFop, PFt, PF, PFm, PFcm) via AF/SLF III (Catani et al., 2005) and pathways between DLPFC and the anterior angular gyrus (PGa) via AF/SLF II (Makris et al., 2005). The frontal-parietal connectivity patterns found in our data are highly consistent with the current view of language pathways (Dick & Tremblay, 2012). There were no direct connections between ventral frontal regions and the parietal areas.

**Table 3:** Group-level fronto-parietal connectivity matrix. Bold font indicates that the connection probability was over 50% (12/24) for group analysis. The individual threshold was set at 2.5%.

		Frontal lobe						Superior parietal lobule							Inferior parietal sulcus			Inferior parietal lobule						
		DLPFC	BA 44	BA 45	BA 47	latOFC	medOFC	5Ci	5M	5L	7PC	7A	7P	7M	IPS1	IPS2	IPS3	PFop	PFt	PF	PFm	PFcm	PGa	PGp
FL	DLPFC		**100**	**100**	**100**	20.8	**100**	33.3	8.3	4.2	**87.5**	20.8	0	16.7	**66.7**	**83.3**	**70.8**	**75.0**	**87.5**	**91.7**	**62.5**	**95.8**	**58.3**	37.5
	BA 44			**100**	**62.5**	4.2	37.5	0	0	0	**66.7**	4.2	0	0	29.2	**58.3**	16.7	**70.8**	**54.2**	**83.3**	25.0	**83.3**	4.2	4.2
	BA 45				**100**	33.3	**100**	0	0	16.7	37.5	8.3	0	0	16.7	29.2	4.2	33.3	12.5	**50.0**	8.3	**50.0**	8.3	0
	BA 47					**100**	**100**	12.5	16.7	20.8	45.8	29.2	16.7	33.3	0	0	16.7	0	0	8.3	0	0	0	12.5
	latOFC						**100**	4.2	0	4.2	8.3	0	0	4.2	0	0	0	0	0	4.2	0	0	0	0
	medOFC							0	0	8.3	29.2	8.3	0	4.2	0	4.2	12.5	0	4.2	0	0	8.3	0	0

SPL	5Ci								**100**	**100**	**100**	**87.5**	41.7	29.2	4.2	8.3	25.0	4.2	8.3	4.2	0	4.2	0	4.2
	5M									**100**	**100**	**100**	**87.5**	**75.0**	12.5	4.2	37.5	0	0	0	0	4.2	0	4.2
	5L										**100**	**100**	**95.8**	45.8	**75.0**	29.2	**100**	4.2	8.3	12.5	4.2	12.5	16.7	**50.0**
	7PC											**100**	**100**	**100**	**100**	**100**	**100**	**100**	**100**	**100**	**91.7**	**100**	**87.5**	**87.5**
	7A												**100**	**100**	**100**	**91.7**	**100**	0	4.2	12.5	25	25.0	**50.0**	**66.7**
	7P													**100**	**70.8**	4.2	**100**	0	0	0	0	0	12.5	45.8
	7M														4.2	0	25.0	0	0	0	0	0	4.2	20.8

IPS	IPS1															**100**	**100**	**54.2**	**79.2**	**100**	**95.8**	**100**	**95.8**	**87.5**
	IPS2																**100**	**100**	**100**	**100**	**91.7**	**100**	**75.0**	**58.3**
	IPS3																	41.7	**70.8**	**87.5**	**91.7**	**95.8**	**95.8**	**95.8**

IPL	PFop																		**100**	**100**	20.8	**100**	20.8	0
	PFt																			**100**	29.2	**100**	37.5	12.5
	PF																				**95.8**	**100**	**75.0**	12.5
	PFm																					**95.8**	**95.8**	33.3
	PFcm																						**79.2**	16.7
	PGa																							**100**
	PGp																							

FL = frontal lobe; SPL = superior parietal lobule; IPL = inferior parietal lobule; DLPFC = dorsolateral prefrontal cortex; BA = Brodmann's areas; medOFC = medial orbitofrontal cortex; latOFC = lateral orbitofrontal cortex; p.Op = pars opercularis; p.Tri = pars triangularis; p.Orb = pars orbitalis; IPS = intraparietal sulcus; 5Ci, 5M, 5L = BA 5 (superior parietal cortex); 7PC, 7A, 7P, 7M = BA 7 (superior parietal cortex); PFop, PFt, PF, PFcm, PFm = supramarginal gyrus; PGa, PGp = angular gyrus.

Fig. 3 was reconstructed based on the averaged tractography results and reveals the major white matter pathways: AF = arcuate fasciculus; SLF = superior longitudinal fasciculus; CB = cingulum bundle; IFOF = inferior fronto-occipital fasciculus; UF = uncinated fasciculus; ILF = inferior longitudinal fasciculus; MdLF = middle longitudinal fasciculus.

### 3.2 Quantification of white matter connectivity using graph-theory network analysis

The second goal of current study was to reveal the underlying topology of the intra/inter-regional structural connectivity for frontal, temporal and parietal lobes. We employed graph-theory network analysis to quantify the network properties in our tractography results (Rubinov & Sporns, 2010). The graph-theory approach is a mathematical framework in which brain is considered as a complex network consisting of nodes reflecting brain regions and edges representing white matter tracts connecting cortical regions (Bullmore & Sporns, 2009). Here, we had 43 nodes (ROIs) and those edges (white matter pathways between ROIs) that survived the 2.5% streamline/50% participant double threshold, resulting in a 43 × 43 adjacency matrix with binary, non-directional connections (See the Supplementary Fig. S2 for the connectivity matrix for the analysis and for more stringent threshold of 5%, see Supplementary Fig. S3.)

**Fig. 4:**
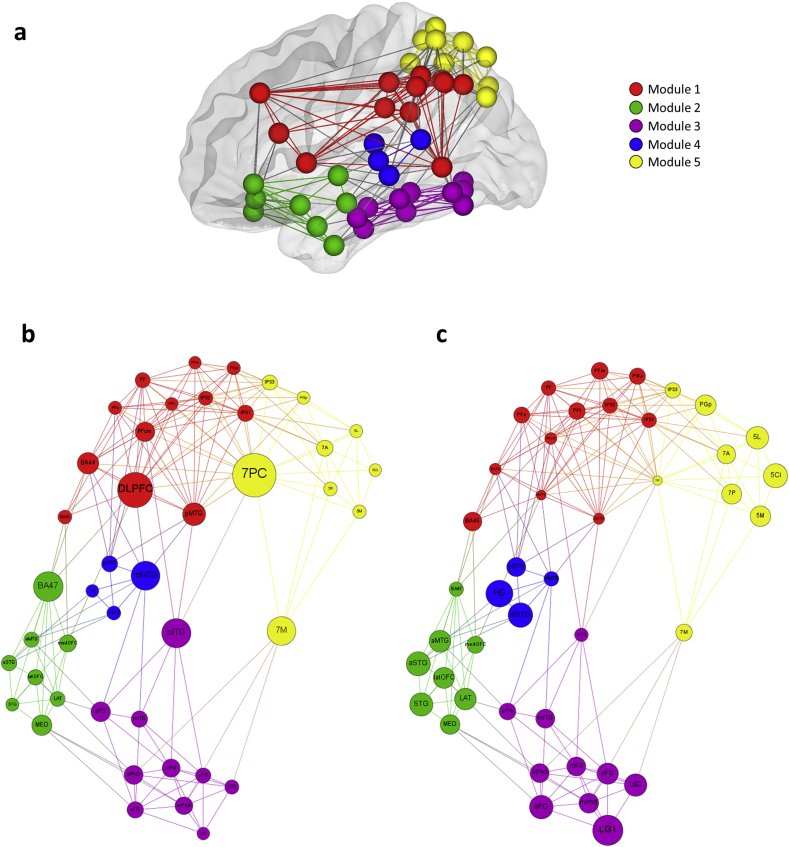
Graphs of the white matter pathways connecting frontal, temporal, and parietal lobe. (A) Graph theory analysis classified the frontotemporal-parietal connectivity into 5 modules. ROIs for the tractography analysis were used as nodes in graph theory analysis. Only significant connections from the tractography analysis were entered as edges for the analysis. Colours in nodes and edges correspond to each module with a unique colour. (B) The result of betweenness centrality analysis. Size of nodes represents the magnitude of betweenness centrality. (C) The result of closeness centrality analysis. Size of nodes represents the magnitude of closeness centrality.

We assessed a global network property – modular structure. Modules have been detected in many complex networks and classify nodes with similar functions by disentangling the structure of the network. The network is divided into modules of nodes with dense connections internally and sparse connections between modules. We applied the optimized algorithm and revealed 5 modules in the network (Fig. 4a). Module 1 was composed of lateral frontal areas (DLPFC, BA44, and BA45), inferior parietal regions (IPC; PFop, PFt, PF, PFm, PFcm, PGa, IPS; IPS1, IPS2), and pMTG in temporal lobe. This frontal-temporal-parietal module corresponds closely to the executive control network (Duncan, 2010). Module 2 clustered OFC and BA 47 in the frontal lobe with temporal polar/anterior regions (STG, lateral temporal pole – LAT, medial temporal pole – MED, aSTG, & aMTG). This orbitofrontal-temporal polar module was strongly connected via the UF and coincides with the social/semantic network (Binney et al., 2012, Olson et al., 2013). Module 3 contained ventral temporal areas inclusively connected via the ILF that reflects the classic visual ‘what pathway’ (Goodale & Milner, 1992). Module 4 represented a sub-network related to auditory processing which consisted of Hechl's gyrus, STG and mMTG. Finally, module 5 contained all SPC regions and 2 additional IPC components (PGp, IPS3), and corresponds to the visuomotor control network (Culham, Cavina-Pratesi, & Singhal, 2006). The strong correspondences between these white-matter defined modules and known cognitive networks are considered in more detail below (Discussion). It was interesting that, although most commonly neighbouring nodes fall into the same module, this was not always the case; the sub-regions of MTG, for example, showed sharp delineations across three different modules. Thus aMTG was the part of module 2, mMTG was in module 5, and pMTG in module 1. In our network, the modularity was .45, which indicates strong community structure (Power, Fair, Schlaggar, & Petersen, 2010).

Fig. 4b illustrates ‘betweenness’ centrality for the nodes within the network, which is a useful measure to quantify how much information passes certain part of a network based on an assumption that optimal paths are used. Therefore, high betweenness centrality identifies nodes that are crucial hubs and/or bridges between modules in a network. In module 1, DLPFC had the highest betweenness centrality followed by BA44 and pMTG. BA 47 was identified as the bridge node in module 2. pITG was the main bridge node linking module 3 with module 1 and 5. In module 4, there were 2 bridge node, 7PC connecting to module 1 and 4 and 7M connecting to module 3 only. mMTG tied module 5 to module 1 and 3.

The other measure of centrality is ‘closeness’, which is defined as the inverse average path length of a node to all other nodes in a network. High closeness centrality represents that the node can reach any other node in the network efficiently and hence plays an important role in integrating the information within the network. The majority of temporal and SPC regions had high closeness values (Fig. 4c). Specifically, LG1 and HG in the temporal lobe showed the highest closeness centrality. Anterior temporal areas (STG, LAT, MED, aSTG, aMTG, aFG) also had high value of the closeness centrality (Table 4).

**Table 4:** The summary of network analysis.

Node	Modularity class	Degree	Closeness centrality	Betweenness centrality	Clustering coefficient
DLPFC	1	16	1.81	116.68	.54
BA44	1	12	1.90	45.73	.61
BA45	1	6	2.33	6.42	.67
BA47	2	10	2.07	88.56	.40
latOFC	2	6	2.48	16.38	.60
medOFC	2	6	2.24	23.80	.60
5Ci	5	4	2.67	0	1.00
5M	5	6	2.45	4.08	.80
5L	5	8	2.57	2.28	.71
7PC	5	20	1.76	160.73	.45
7A	5	11	2.33	19.40	.58
7P	5	7	2.43	5.35	.76
7M	5	7	2.21	83.30	.43
IPS1	1	15	2.10	18.97	.64
IPS2	1	14	2.12	8.98	.73
IPS3	5	13	2.14	13.94	.65
PFo	1	10	2.19	3.78	.87
PFt	1	10	2.21	.53	.91
PF	1	14	2.10	12.59	.69
PFm	1	9	2.24	.10	.97
PFcm	1	15	1.93	35.78	.62
PGa	1	11	2.19	4.39	.82
PGp	5	8	2.45	1.92	.82
STG	2	6	2.62	3.47	.73
LAT	2	7	2.52	11.95	.52
MED	2	8	2.40	40.25	.46
aSTG	2	6	2.67	15.76	.40
aMTG	2	6	2.52	13.27	.40
aITG	3	7	2.21	36.83	.38
aFG	3	8	2.64	19.96	.50
aPhG	3	7	2.36	32.73	.48
HG	4	3	2.83	1.12	.67
mSTG	4	5	2.69	6.98	.50
mMTG	4	9	2.10	84.50	.28
mITG	3	6	2.36	19.74	.60
mFG	3	9	2.36	28.50	.53
mPhG	3	8	2.40	26.84	.57
pSTG	4	7	2.36	17.89	.62
pMTG	1	15	1.88	53.39	.58
pITG	3	7	2.02	86.64	.38
pFG	3	7	2.50	14.40	.62
LG1	3	5	3.05	.20	.90
LG2	3	6	2.57	8.88	.67

DLPFC = dorsolateral prefrontal cortex; BA = Brodmann's areas; medOFC = medial orbitofrontal cortex; latOFC = lateral orbitofrontal cortex; p.Op = pars opercularis; p.Tri = pars triangularis; p.Orb = pars orbitalis; IPS = intraparietal sulcus; 5Ci, 5M, 5L = BA 5 (superior parietal cortex); 7PC, 7A, 7P, 7M = BA 7 (superior parietal cortex); PFop, PFt, PF, PFcm, PFm = supramarginal gyrus; PGa, PGp = angular gyrus; STG = superior temporal gyrus; LAT = lateral temporal pole; MED = medial temporal pole; MTG = middle temporal gyrus; ITG = inferior temporal gyrus; FG = fusiform gyrus; PhG = parahippocampal gyrus; HG = Heschl's gyrus; LG1 = lingual gyrus next to fusiform gyrus; LG2 = medial lingual gyrus; a = anterior temporal; m = middle temporal; p = posterior temporal.

## 4 Discussion

The two key objectives of this investigation were (a) to map the detailed, large-scale white-matter connectivity between 43 temporal, parietal and ventral/lateral frontal areas, and then (b) to utilise graph-theory analysis to quantify the network properties of this large-scale connectome, which covers the human tertiary association cortices, critical for various higher cognitive functions. The Discussion is split, therefore, into two sections in order to consider not only these large-scale anatomical results but also the direct relationship between the five structural modules, identified in this study, and various core functional networks that are repeatedly observed in ICA investigations of functional and resting-state fMRI.

### 4.1 A large-scale tractography interconnecting frontal, temporal, and parietal lobes

A key general finding from this study was that there is a strong contrast in the nature of intra- versus inter-lobe connectivity. For all three lobes, intra-area connectivity was high and generally graded in nature (presumably reflecting local U-shaped fibres) with few, if any, sharp divisions in the observed connectome. In contrast, the inter-lobe connectivity was relatively discrete and regionally-specific such that only small sub-regions exhibited long-range connectivity to another lobe. The functional consequences of this contrastive connectivity profile can be predicted from previous computational models which have constrained the model architecture with connectivity information (Lambon Ralph et al., 2001, Plaut, 2002, Ueno et al., 2011): the graded intra-lobe connectivity is consistent with primarily-similar local computations, such that the region as a whole has the properties of mass action and graded degradation after damage (Farah and Mcclelland, 1991, Plaut, 2002). Whilst still partaking in the same basic local computation, the function of some sub-regions will be additionally influenced by long-range connections leading to graded, partial specialisation (Lambon Ralph et al., 2001, Plaut, 2002). Indeed, as noted in the Introduction and by a variety of authors (Alvarez and Squire, 1994, Binder et al., 1997, Seeley et al., 2007), many higher cognitive functions or activities seem to reflect the joint action of multiple, distributed brain regions. The long-range connectivity observed in this study is consistent with the notion that these higher cognitive activities require the synchronised combination of various primary domain-general computations (e.g., working memory requires registration of information, short-term maintenance and interaction with executive mechanisms; picture naming requires decoding and recognition of a visual stimulus, activation of its meaning and, in turn, of the speech production system).

Our detailed exploration of the temporal lobe replicates and extends previous explorations of the rostral half of the temporal lobe (Binney et al., 2012, Fan et al., 2014) to the remainder of the temporal lobe by adding a posterior cross-section covering the traditional pMTG area and occipitotemporal junction regions. As found previously, there is a continuous yet graded pattern of connectivity within the temporal lobe such that each area is connected to its lateral and anterior-posterior neighbours. This type of graded connectivity provides the basis for informational convergence which is maximal in the lateral and polar temporal regions – consistent with the role of these regions in multimodal semantic processing (Binney et al., 2012, Lambon Ralph, 2014, Visser et al., 2012). The only contrasting areas are the superior temporal and Heschl's gyri which have only one neighbouring gyrus, and thus their (acoustic processing) function can remain relatively modality-specific (Binney et al., 2012) and thus preserve functional “fidelity” (Mesulam, 1998).

The parietal lobe plays a critical role in integrating sensory information from various modalities and numerous cognitive functions. As the parietal cortex is involved in many different cognitive functions, many researcher have attempted to parcellate its function and structure (Caspers et al., 2006, Nelson et al., 2010). However, a recent fMRI meta-analysis study demonstrated that all sub-regions were engaged in various tasks covering various cognitive domains (Humphreys & Lambon Ralph, 2014). Specifically, there was a major dorsal versus ventral functional division of a domain-general nature, with the dorsal regions involved in tasks that require executive control whilst the ventral areas were implicated in more automatic processes across domains (Corbetta & Shulman, 2002). Our tractography goes beyond previous functional connectivity explorations and indicates that these function characteristics reflect the core underlying white-matter connectivity: the domain-general nature of the entire region could follow from its high-level of interconnectivity across all sub-regions (Fig. 2c); and the dorsal-ventral variation could reflect the differential connectivity of lateral prefrontal regions to 7PC, IPS and superior SMG sub-regions (which together form the ‘multi-demand network’: Duncan, 2010): whilst there is no evidence of this connectivity to ventral areas (e.g., AG).

The frontal lobe has been considered a key region in higher-order cognitive control (Duncan and Owen, 2000, Miller and Cohen, 2001, Petrides, 1996). One view of its functional organisation suggests that the components of the control process fall along two axes: the rostral-caudal axis and the dorsal-ventral axis (Petrides, 2005). For example, action control is implemented in the frontal region along the rostral-caudal axis, with the more posterior regions implicated in the simpler actions and representations (Koechlin et al., 2003, Petrides, 2005), whereas the dorsal-ventral axis is related to two distinctive levels of control: monitoring information in the dorsolateral area (which connects to dorsal IPL/SPL sub-regions to form the multi-demand network) and decision making in the ventrolateral area (Petrides, 2005). Again, this functional distinction potentially relies on its inter-lobe connectivity patterns (Petrides, 2005, Thiebaut de Schotten et al., 2012, Yeterian et al., 2012). Within the prefrontal cortex, the sub-regions were highly interconnected with each other via short U-shape fibres. This pattern of the intra-lobe connectivity suggests the control processes will be graded along both axes (Catani et al., 2012, Petrides, 2005).

Moving beyond each lobe in isolation, our tractography results revealed various distinctive inter-lobe connections. The temporal lobe divides into six sub-sections with distinctive connections to the frontal and parietal areas: (1) temporopolar regions connect to OFC and BA 47 via UF exclusively; (2) anterior temporal STG and MTG connect to BA 47 via the IFOF; (3) dorsal-posterior temporal areas were linked with lateral frontal regions and most of IPC via the AF dorsally and MdLF/parietal branch of IFOF, ventrally; (4) basal-medial temporal areas have a direct connection to precuneus via the CB; (5) ventrolateral regions display an absence of connections to the other lobes; (6) pMTG and pITG share a pathway to the lateral bank of SPC. Fronto-parietal connections also exhibit regional distinctive patterns of connectivity: (a) DLPFC and BA 44 were linked with the lateral bank of SPC via SLF I; (b) AF/SLF III connected frontal regions to supramarginal gyrus and IPS; (c) DLFPC also had direct connections with angular gyrus via AF/SLF II; (d) there was an absence of connectivity between OFC and parietal lobules.

Overall, the inter-lobe tractography described here is in line with previous findings using post-mortem axonal tracing in monkeys, post-mortem brain dissection and *in vivo* tractography in human. UF has been well described in its anatomy, connecting ventrolateral frontal cortices with temporopolar cortex (Gloor, 1997). However, the functions of UF are still not clear but might include social processing by linking ventral frontal and medial temporal limbic regions and/or semantic cognition by connecting anterior temporal semantic representational systems with executive mechanisms supported by ventrolateral frontal areas (Binney et al., 2012, Olson et al., 2013). IFOF is a long white-matter bundle with multiple branches that connects occipital cortex, temporal areas, ventrolateral frontal cortex and inferior parietal regions (Martino et al., 2010, Schmahmann et al., 2007). This tract has been debated both with regard to its anatomy and function but recent studies suggest that IFOF is consistent with the ventral language pathway (Parker et al., 2005) and/or a part of controlled semantic processes (Duffau et al., 2005).

The CB is a C-shape structure projecting from cingulate cortex to entorhinal cortex: the anterior part of this pathway is linked with frontal cortices and the posterior part is specifically connected with parahippocampal gyrus (Mufson & Pandya, 1984). Functionally, the anterior cingulum is implicated in emotion and cognitive control, whereas the posterior cingulum has been related to episodic memory, attention, and spatial navigation (Shannon & Buckner, 2004). Consistent with these previous findings, our tractography demonstrated a direct link between the parahippocampal gyrus and precuneus (Mufson & Pandya, 1984), and its emergent functional role by connecting two major areas associated with episodic memory functions.

The AF and SLF have been considered to be an important language pathway. Recently, there have been attempts to distinguish branches of AF/SLF. For example, Catani et al. (2005) delineated AF/SLF into three segments, a direct temporo-frontal segment and two indirect temporal-parietal-frontal segments. Recent non-human primate data have suggested four distinctions: SLF I, SLF II, SLF III, and AF (Schmahmann and Pandya, 2006, Schmahmann et al., 2007). Our results demonstrated dissociable pathways attributable to each of these four subcomponents: (1) DLPFC and BA 44 linking with the lateral bank of SPC via SLF I; (2) DLPFC also had direct connections with angular gyrus via SLF II; (3) Inferior frontal regions to supramarginal gyrus and IPS via SLF III; (4) Dorsal-posterior temporal areas connecting with lateral frontal regions and IPC via AF.

Additionally, our tractography demonstrated two direct pathways connecting the posterior temporal regions to the parietal lobe. Martino et al. (2010) found a dorsal subcomponent of IFOF was terminated into the convexity surface of SPL. A direct pathway linking the posterior temporal regions to posterior parietal lobule has been verified and attributed to either MdLF (Seltzer & Pandya, 1984) or ILF (Schmahmann et al., 2007). Thus, the pathways found here might be a parietal branch of IFOF to SPL and a parietal branch of ILF to IPL.

### 4.2 Networks emerging from the patterns of white matter connectivity

Whilst of interest by itself (see section above), by obtaining a large-scale detailed connectome for 43 areas within temporal, parietal and ventral-lateral frontal regions, we were then able to quantify the network characteristics within this web of white-matter connections. Strikingly, the five derived physically-connected sub-networks seem to correspond directly to, and might be the basis of, five known functional networks (as identified repeatedly in analyses of resting-state fMRI).

We are not, of course, the first to suggest a direct relationship between white-matter connections and brain functions – but as far as we are aware this is the first study which has the necessary large-scale baseline data required to explore the relationship between connectivity across tertiary association cortices and higher cognitive functions. The relationship between structure and function was noted by Brodmann (1909) himself who suggested that the pattern of interconnections was likely to have a large influence on function (something he referred to as “fibrilloarchitectonics”) but he was unable to explore this with the techniques available at that time. It is only since the rise of MRI-based techniques to infer human white-matter connectivity *in vivo* that researchers have begun to explore the relationship between functional networks and the underlying structural networks. By focussing on anatomically detailed sub-divisions of frontal, temporal, and parietal areas, we were able to extract functional-structural parallels for five higher cortical networks.

The finding of a frontal-temporal-parietal network is in line with previous studies. As noted above, Duncan (2010) has proposed a multiple demand system based on a frontoparietal network including prefrontal cortex, inferior frontal sulcus, anterior cingulate/presupplementary motor area and IPS. Module 1 found in our tractography is highly overlapping with this frontoparietal multiple demand network except that it also includes the pMTG. Although not the focus of previous descriptions and studies, both fMRI studies of executive functions (Nee et al., 2013) and ICA investigations of resting-state fMRI have included pMTG within the same network (Spreng, Sepulcre, Turner, Stevens, & Schacter, 2013). In addition, a recent meta-analysis study demonstrated that pMTG was a component of the control network in comparison of higher versus low semantic control demands (Noonan, Jefferies, Visser, & Lambon Ralph, 2013). Moreover, pMTG has been observed to have strong anatomical and functional connections with the rest of the frontoparietal network (Catani et al., 2005, Saur et al., 2008).

The second module represents an OFC-TP network exclusively mediated via UF. Temporopolar areas (ATL) and ventrolateral prefrontal cortex have been implied in semantic cognition (Binney et al., 2012) and various aspects of social cognition, including OFC's role in emotion, decision-making and expectation (Bechara, Damasio, & Damasio, 2000) as well as the involvement of superior ATL regions in social knowledge and theory of mind (Olson et al., 2013). These are not mutually exclusive possibilities as processing and manipulation of social stimuli may be based on semantic knowledge more generally. Alternatively, the two functions may reflect the two sub-branches of UF, with medial ‘limbic’ anterior temporal areas connected to OFC (for social-emotion processing), and more polar-lateral temporal regions linking to ventral inferior prefrontal cortex (for semantic cognition).

The basal-temporal module 3, contains medial, ventral and inferior temporal regions, and has been associated with three representational systems. Classically, the ventrolateral temporal areas support the visual “what pathway”, key for object processing and recognition (Goodale & Milner, 1992). More recently, the ventral anterior temporal region (at the apex of the ventral visual stream) has been demonstrated to be involved in multimodal semantic processing (Shimotake et al., 2015), which is consistent with a posterior-to-anterior hierarchical processing stream. Medially, the parahippocampal regions are crucial for episodic memory (Alvarez & Squire, 1994) and visuospatial processing (Epstein & Kanwisher, 1998). More recent studies have noted the strong visual input and influence on contextual associative processing in the parahippocampal cortex including the spatial relations between objects and their surroundings and certain expected behaviours in those environments (Aminoff et al., 2013, Murray et al., 2007).

The auditory module 4 consists of Hechl's gyrus and neighbouring mSTG, pSTG and mMTG. The primary auditory network is a part of the auditory system, performing basic and higher functions in hearing (Zatorre, Belin, & Penhune, 2002). Using the same methodology, it has been demonstrated that visual, auditory and sensory-motor systems were clustered according to their functions by manifesting the evolutionary optimization of brain structure and function (Hilgetag, Burns, O'Neill, Scannell, & Young, 2000). Our analysis also delineated the primary auditory network from the lateral associative network, thereby revealing the structural optimization in the primary sensory function.

The visuomotor control network, module 5, includes all SPC areas, IPS3 and PGp. Traditionally, this network has been associated with the visual “where pathway” and recently with an important role in visuomotor control such as multimodal encoding of location, reaching, grasping and eye movements (Culham et al., 2006, Goldenberg and Spatt, 2009). The area, 7PC, was found to be the hub of this network bridging to the executive control network (module 1) and representation network (module 3). 7PC is the lateral bank of SPC located immediately above IPS and has been associated with many cognitive domains. A recent fMRI meta-analysis revealed that this region was activated in a range of cognitive activities including top-down attention, numerical processing, executive semantics, phonological tasks and tool-related functions (Humphreys & Lambon Ralph, 2014). Thus, we suggest that 7PC may be a domain-general area within SPC, consistent with its anatomical characteristic as a critical hub linking to frontal and temporal lobes.

### 4.3 Methodological considerations and limitations of the current study

In tractography, there are multiple sources of error to validate the identified fibre pathways due to the issues of track reconstruction such as partial volume effect, the branching of fibre pathways and the length and shape of paths tracked. As a results, there is a level of uncertainty in any tractographic data, including both false positive (Type I) and false negative (Type II) errors. However, recent studies have made significant advances to ameliorate tractographic methodologies for modelling complex fibre orientations and sampling the uncertainty in fibre orientation (Behrens et al., 2007, Chung et al., 2006, Haroon et al., 2009, Lazar and Alexander, 2005). Specifically, the current study employed a sophisticated combination of probabilistic tractography using PICo (Parker, Haroon, & Wheeler-Kingshott, 2003) and CSD (Tournier et al., 2007, Tournier et al., 2008) to overcome these issues, thereby increasing the anatomical accuracy and validity of the white matter pathways. PICo takes into account the local uncertainty in fibre orientation by running the streamline process repeatedly and generates probabilistic maps of connectivity (Behrens et al., 2003, Parker et al., 2003). Also, PDFs generated using the CSD method (Tournier et al., 2007, Tournier et al., 2008) estimate the distribution of possible fibre orientation based on the assumption that all fibre populations share identical diffusion characteristics. Consequently, partial volume effects can be described by differences in anisotropy. A spherical function from the CSD provides the fibre orientation distribution (FOD), illustrating the number and direction of the orientations within a given voxel and their relative weightings. Model-based residual bootstrapping enables to sample the FOD by obtaining an estimate of the uncertainty in fibre orientations (Chung et al., 2006, Haroon et al., 2009). Accordingly, these methods have demonstrated their efficacy and superiority in resolving narrow crossing fibre angles (e.g., 30°) (Tournier et al., 2008) and producing robust and reproducible tracking results (Jeurissen et al., 2011). The combination of the probabilistic tractography and CSD methods implemented in the current study successfully delineated the white matter connectivity of brain regions including the insula (Cloutman et al., 2012), inferior parietal regions (Cloutman, Binney, Morris, Parker, & Lambon Ralph, 2013), and the temporal lobe (Bajada et al., 2016, Binney et al., 2012).

Although probabilistic tractography techniques have made substantial advancements, important limitations still remain which need to be considered in interpreting any tractography results (Jbabdi & Johansen-Berg, 2011). The key limitations of relevance to the current study are the issues of distance effect and thresholding (Jones, 2008, Morris et al., 2008). Each step of the propagation of a pathway has a degree of uncertainty in fibre orientation and the accumulation of this uncertainty from voxel to voxel leads to a decrease in connection probability with increasing distance between regions (Morris et al., 2008). As a result, it is difficult to track long-range connections and to interpret tracking results because the connection probability is not uniform across distance. In addition, it is difficult to determine a threshold value that successfully identify true connectivity, with minimising the rate of both false positives (Type I errors in regions close to the seed) and false negatives (Type II errors in more distant regions). As there is no consensus regarding this issue, the current study took a conservative approach for thresholding. As described in Materials and methods, streamline density was used to define a threshold value by taking the average of the connectivity distribution across the whole brain, reflecting values from regions with both short and long connectivity distances. Through this thresholding, we most likely achieved a conservative cut-off for longer pathways which produced fewer false positives in the long range connections and fibre pathways identified. Despite of this conservative thresholding, it is noted that there may be long-range connections left undetected in the current study.

The current study utilized *in vivo* probabilistic tractography to explore the white matter connectivity between associative cortices, identified a number of pathways consistent with previous primate and human dissection studies, and quantified the network properties of this large-scale connectome by employing the graph theory. We acknowledge that further studies may be needed to support and validate our findings, such as clinical correlations or fMRI data.
