# Supplementary material for: The structural connectivity of higher order association cortices reflects human functional brain networks
Source: Cortex. 2017 Dec;97:221–39. doi: 10.1016/j.cortex.2016.08.011 (PMC5726605; doi:10.1016/j.cortex.2016.08.011)
Supplement: Supplementary file 1 [file mmc1.docx]

Organisation of supplementary information

**SI Results**

a. Figure S2 – this relates to Figures 1 & 2: it shows the stability of the results when a different threshold is applied to the tractography data.

b. Figure S1. The group connectivity matrix for graph theory analysis.

c. Figure S3 - this relates to Figure 4: it shows the stability of the graph theory analysis when a different threshold is applied to the tractography data.


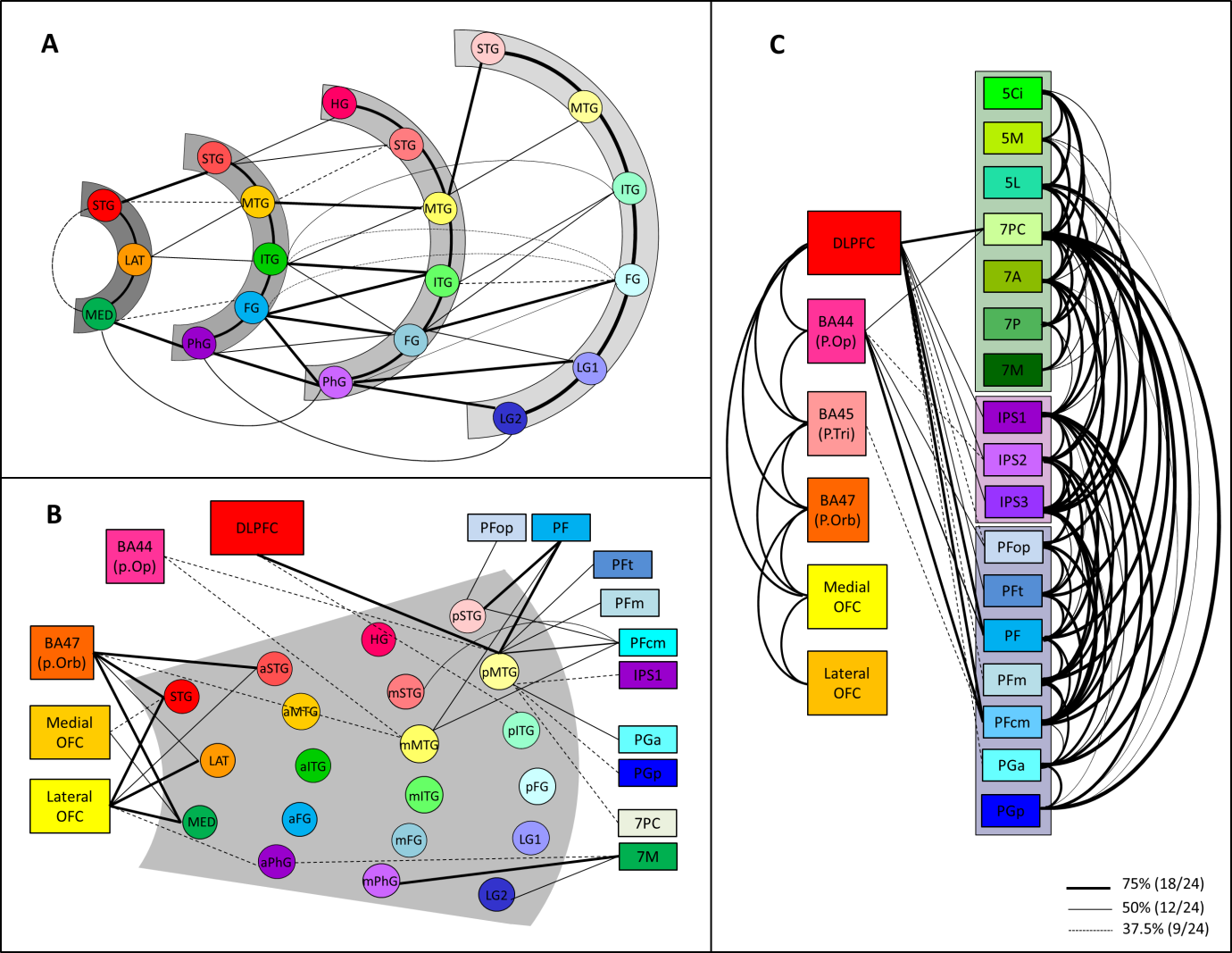


Figure S1. The tractography results thresholded at 5% for individual level. (A) Intratemporal lobe connectivity. The temporal lobe regions within each slice are represented by circles. (B) Extratemporal lobe connectivity. Temporal lobe regions are represented as circles within the grey part. The frontal and parietal regions are represented as boxes, each with a unique colour. (C) Fronto-parietal lobe connectivity. Connecting lines illustrate white matter pathways between pairs of regions survived at the thresholded connectivity matrix. Thick lines represent white matter pathways thresholded at 75%, thin lines thresholded at 50%, and dash lines thresholded at 37.5% from group analysis. STG = superior temporal gyrus; LAT = lateral temporal pole; MED = medial temporal pole; MTG = middle temporal gyrus; ITG = inferior temporal gyrus; FG = fusiform gyrus; PhG = parahippocampal gyrus; HG = Heschl’s gyrus; LG1 = lingual gyrus next to fusiform gyrus; LG2 = medial lingual gyrus; DLPFC = dorsolateral prefrontal cortex; BA = Brodmann’s areas; OFC =orbitofrontal cortex; p.Op = pars opercularis; p.Tri = pars triangularis; p.Orb = pars orbitalis; IPS =intraparietal sulcus; 5Ci, 5M, 5L = BA 5 (superior parietal cortex); 7PC, 7A, 7P, 7M = BA 7 (superior parietal cortex); PFop, PFt, PF, PFcm, PFm = supramarginal gyrus; PGa, PGp = angular gyrus; STG = superior temporal gyrus; LAT = lateral temporal pole; MED = medial temporal pole; MTG = middle temporal gyrus; ITG = inferior temporal gyrus; FG = fusiform gyrus; PhG = parahippocampal gyrus; HG = Heschl’s gyrus; LG1 = lingual gyrus next to fusiform gyrus; LG2 = medial lingual gyrus; a = anterior temporal; m = middle temporal; p = posterior temporal


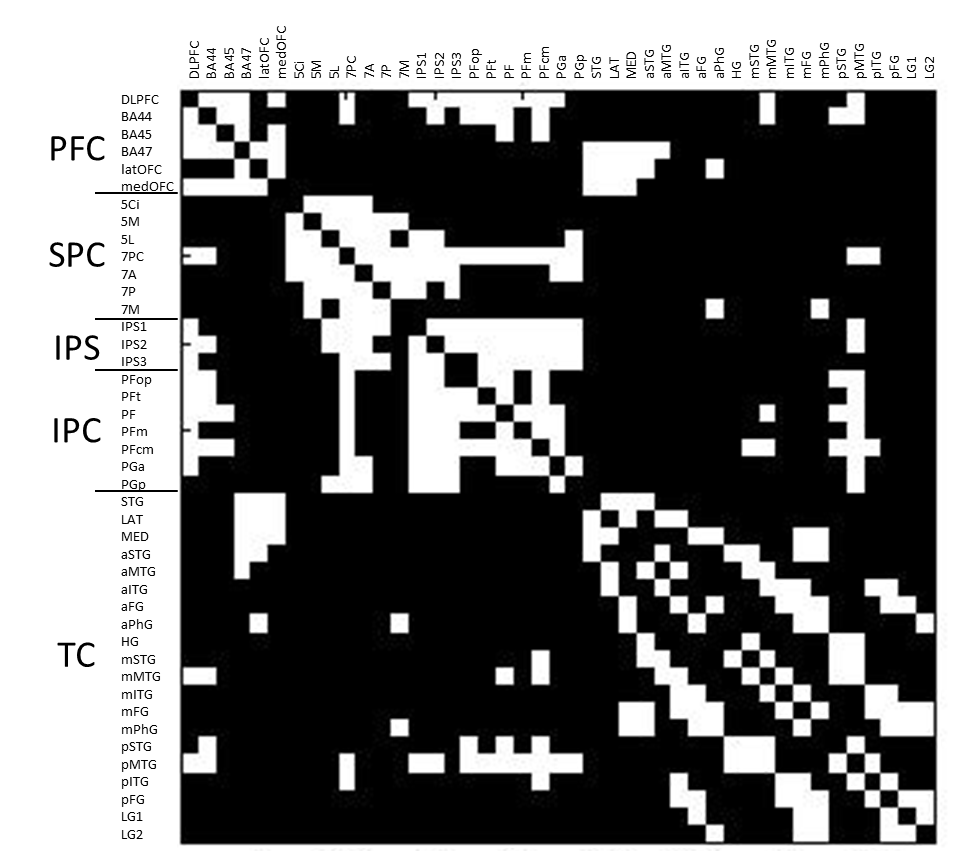


Figure S2. The group connectivity matrix for graph theory analysis. White colour represents where there is significant probability of connection between regions.


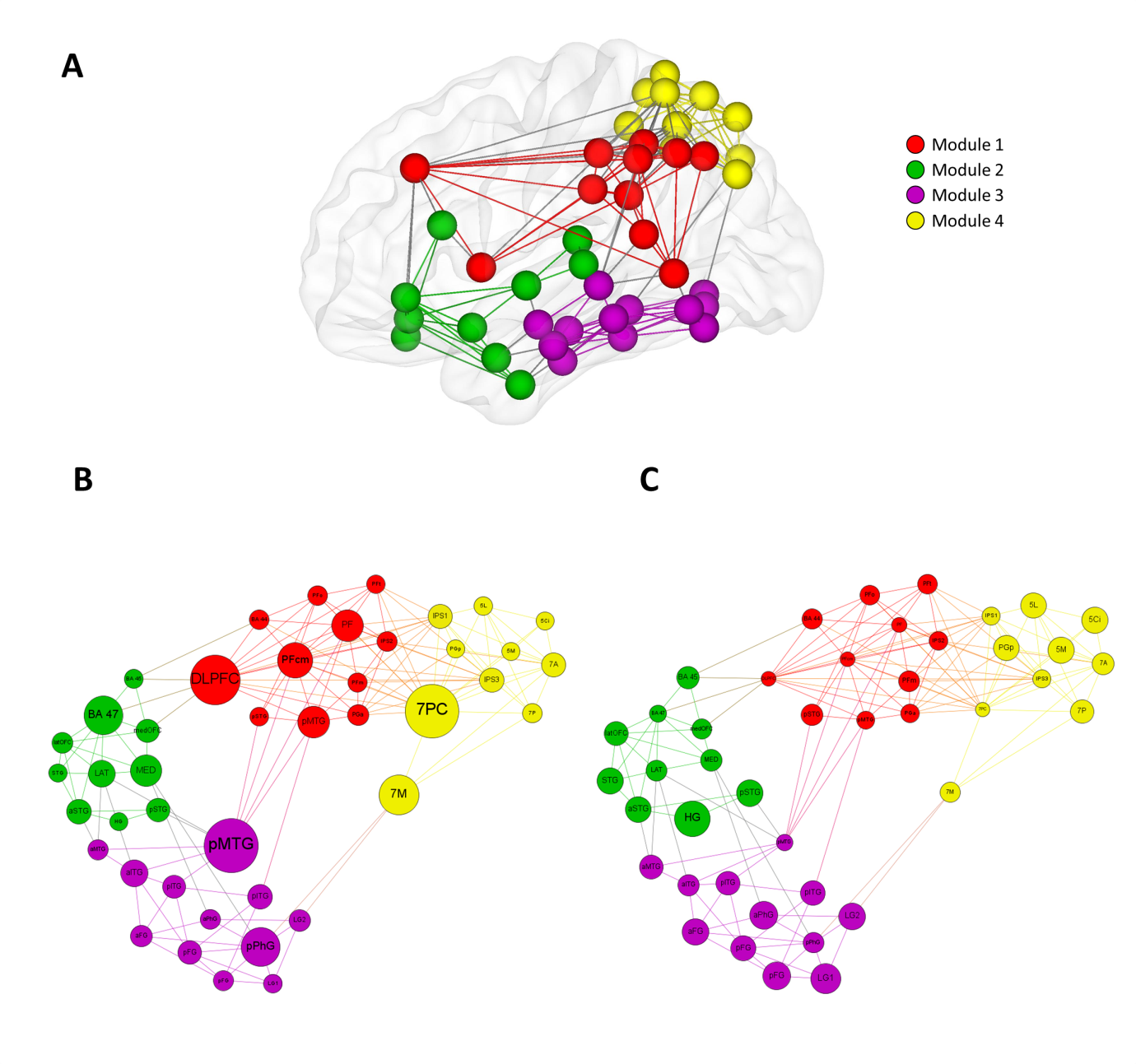
Figure S3. Graphs of the white matter pathways connecting frontal, temporal, and parietal lobe (thresholded at 5% for individual level). (A) Graph theory analysis classified the frontotemporal-parietal connectivity into 4 modules. (B) The result of betweenness centrality analysis. Size of nodes represents the magnitude of betweenness centrality. (C) The result of closeness centrality analysis. Size of nodes represents the magnitude of closeness centrality. DLPFC = dorsolateral prefrontal cortex; BA = Brodmann’s areas; medOFC = medial orbitofrontal cortex; latOFC = lateral orbitofrontal cortex; p.Op = pars opercularis; p.Tri = pars triangularis; p.Orb = pars orbitalis; IPS = intraparietal sulcus; 5Ci, 5M, 5L = BA 5 (superior parietal cortex); 7PC, 7A, 7P, 7M = BA 7 (superior parietal cortex); PFop, PFt, PF, PFcm, PFm = supramarginal gyrus; PGa, PGp = angular gyrus; STG = superior temporal gyrus; LAT = lateral temporal pole; MED = medial temporal pole; MTG = middle temporal gyrus; ITG = inferior temporal gyrus; FG = fusiform gyrus; PhG = parahippocampal gyrus; HG = Heschl’s gyrus; LG1 = lingual gyrus next to fusiform gyrus; LG2 = medial lingual gyrus; a = anterior temporal; m = middle temporal; p = posterior temporal
